# The Curing Coma Campaign International Survey on Coma Epidemiology, Evaluation, and Therapy (COME TOGETHER)

**DOI:** 10.1007/s12028-021-01425-8

**Published:** 2022-02-09

**Authors:** Raimund Helbok, Verena Rass, Ettore Beghi, Yelena G. Bodien, Giuseppe Citerio, Joseph T. Giacino, Daniel Kondziella, Stephan A. Mayer, David Menon, Tarek Sharshar, Robert D. Stevens, Hanno Ulmer, Chethan P. Venkatasubba Rao, Paul Vespa, Molly McNett, Jennifer Frontera, the Curing Coma Campaign and its Contributing Members

**Affiliations:** 1 Department of Neurology, Neuro-Intensive Care Unit, Medical University of Innsbruck, Anichstrasse 35, 6020 Innsbruck, Austria; 2 Department of Neuroscience, Istituto di Ricerche Farmacologiche Mario Negri IRCCS, Milan, Italy; 3 Department of Physical Medicine and Rehabilitation, Spaulding Rehabilitation Hospital, Harvard Medical School, Harvard University, Boston, MA USA; 4 Center for Neurotechnology and Neurorecovery, Department of Neurology, Massachusetts General Hospital, Boston, MA USA; 5 Neuro-Intensive Care, ASST Di Monza, Monza, Italy; 6 School of Medicine and Surgery, Università Milano Bicocca, Milan, Italy; 7 Department of Neurology, Rigshospitalet, Copenhagen University Hospital and Faculty of Health and Medical Sciences, University of Copenhagen, Copenhagen, Denmark; 8 Department of Neurology, New York Medical College, Valhalla, NY USA; 9 Division of Anaesthesia, University of Cambridge, Cambridge, UK; 10 Neuro-Intensive Care Medicine, Sainte-Anne Hospital, University of Paris, GHU-Psychiatry & Neurosciences, Paris, France; 11 Departments of Anesthesiology and Critical Care Medicine, Neurology, and Neurosurgery, School of Medicine, Johns Hopkins University, Baltimore, MD USA; 12 Director Department of Medical Statistic, Informatics and Health Economics, Medical University of Innsbruck, Innsbruck, Austria; 13 Division of Vascular Neurology and Neurocritical Care, Baylor College of Medicine and CHI Baylor St Luke’s Medical Center, Houston, TX USA; 14 Departments of Neurology and Neurosurgery, David Geffen School of Medicine, University of California, Los Angeles, Los Angeles, CA USA; 15 College of Nursing, The Ohio State University, Columbus, OH USA; 16 Department of Neurology, Grossman School of Medicine, New York University, New York, NY USA

**Keywords:** Coma, Disorders of consciousness, Critical care, Survey

## Abstract

**Background:**

Although coma is commonly encountered in critical care, worldwide variability exists in diagnosis and management practices. We aimed to assess variability in coma definitions, etiologies, treatment strategies, and attitudes toward prognosis.

**Methods:**

As part of the Neurocritical Care Society Curing Coma Campaign, between September 2020 and January 2021, we conducted an anonymous, international, cross-sectional global survey of health care professionals caring for patients with coma and disorders of consciousness in the acute, subacute, or chronic setting. Survey responses were solicited by sequential emails distributed by international neuroscience societies and social media. Fleiss *κ* values were calculated to assess agreement among respondents.

**Results:**

The survey was completed by 258 health care professionals from 41 countries. Respondents predominantly were physicians (*n* = 213, 83%), were from the United States (*n* = 141, 55%), and represented academic centers (*n* = 231, 90%). Among eight predefined items, respondents identified the following cardinal features, in various combinations, that must be present to define coma: absence of wakefulness (81%, *κ* = 0.764); Glasgow Coma Score (GCS) ≤ 8 (64%, *κ* = 0.588); failure to respond purposefully to visual, verbal, or tactile stimuli (60%, *κ* = 0.552); and inability to follow commands (58%, *κ* = 0.529). Reported etiologies of coma encountered included medically induced coma (24%), traumatic brain injury (24%), intracerebral hemorrhage (21%), and cardiac arrest/hypoxic-ischemic encephalopathy (11%). The most common clinical assessment tools used for coma included the GCS (94%) and neurological examination (78%). Sixty-six percent of respondents routinely performed sedation interruption, in the absence of contraindications, for clinical coma assessments in the intensive care unit. Advanced neurological assessment techniques in comatose patients included quantitative electroencephalography (EEG)/connectivity analysis (16%), functional magnetic resonance imaging (7%), single-photon emission computerized tomography (6%), positron emission tomography (4%), invasive EEG (4%), and cerebral microdialysis (4%). The most commonly used neurostimulants included amantadine (51%), modafinil (37%), and methylphenidate (28%). The leading determinants for prognostication included etiology of coma, neurological examination findings, and neuroimaging. Fewer than 20% of respondents reported routine follow-up of coma survivors after hospital discharge; however, 86% indicated interest in future research initiatives that include postdischarge outcomes at six (85%) and 12 months (65%).

**Conclusions:**

There is wide heterogeneity among health care professionals regarding the clinical definition of coma and limited routine use of advanced coma assessment techniques in acute care settings. Coma management practices vary across sites, and mechanisms for coordinated and sustained follow-up after acute treatment are inconsistent. There is an urgent need for the development of evidence-based guidelines and a collaborative, coordinated approach to advance both the science and the practice of coma management globally.

**Supplementary Information:**

The online version contains supplementary material available at 10.1007/s12028-021-01425-8.

## Introduction

Coma is widely encountered throughout health care settings and may occur in the context of a variety of different acute neurological disorders [1–3]. Although definitions of coma have been proposed [4], many are within the context of disease-specific conditions, such as cardiac arrest, traumatic brain injury, and stroke [5], resulting in a variety of operational indices to document its presence and severity. Moreover, the clinical definition of “unarousable unresponsiveness,” as proposed by Plum and Posner, is being challenged by recent developments using advanced imaging and electrophysiologic tools [6]. Similarly, assessment modalities, diagnostic and management practices, and approaches to prognostication among comatose patients may differ depending on underlying diagnosis, treatment setting, and availability of resources in acute and postacute phases [7]. To advance the science and practice management of comatose patients worldwide, a comprehensive and collaborative approach is needed to establish common diagnostic criteria, data elements, assessments and therapies, and care coordination throughout initial and long-term phases of coma recovery.

In response to this need, the Neurocritical Care Society (NCS) established the Curing Coma Campaign (CCC) [5, 8]. The overarching goal of the CCC is to bridge disorders of consciousness (DoC) science with patient-facing resources to improve outcomes and quality of life for patients and families dealing with coma and other DoC (e.g., unresponsiveness wakefulness syndrome, minimally conscious state). Ultimately, the intent is to develop an enduring framework for studying mechanisms of coma, promoting awareness, and developing evidence-based treatments for patients with acute illness who develop coma [9–12]. An initial step toward these efforts is establishing baseline metrics on current clinical practices across disciplines, settings, populations, and causes.

Therefore, the primary objective of the Coma Epidemiology, Evaluation, and Therapy (COME TOGETHER) survey was to assess international variability of defining coma clinical features and etiology and to identify current practices for diagnosis, management, and prognosis of comatose patients across underlying disease and mechanisms of coma. Specific aims included (1) assessing the agreement on predefined cardinal features of coma, (2) determining common etiologies of acute coma in intensive care units (ICUs) worldwide, (3) quantifying assessment tools and treatment strategies used by a global network of clinicians in the management of comatose patients admitted to the ICU, and (4) determining approaches to prognostication in comatose patients.

## Methods

### Study Design and Ethical Approval

We designed an international cross-sectional online survey to assess variability in coma definitions, etiologies, treatment strategies, and prognosis among health care professionals who care for comatose patients. The COME TOGETHER survey was designed by the Prospective Studies Working Group of the CCC. The Prospective Studies Working Group consisted of 14 clinicians (physicians, neuropsychologists, nurses, and physicians assistants) and neuroscientists with expertise in DoC. The working group represented 13 international academic medical centers from the fields of neurology, neurosurgery, physical medicine and rehabilitation, nursing, and neuroscience (Supplemental Table 2). The group met regularly between September 2019 and July 2020 to identify gaps in the literature regarding coma care and develop the study protocol and corresponding survey items. The conduct of the survey was approved by the Ethics Committee of the Medical University of Innsbruck (EK-1078/2020) and endorsed by the NCS and the CCC.

### Participants

The target audience was health care professionals caring for patients with coma and DoC in the acute, subacute, or chronic setting. Multiple responses from different clinicians at the same institution were allowed. There were no exclusion criteria. There were no incentives or marketing materials.

### Survey Distribution

The survey was launched on September 9, 2020, and open through January 18, 2021. Participants were recruited through blast emails distributed by international neuroscience societies, including the NCS, as well as through promotion during scientific meetings and on social media. Study data were collected and managed by using Research Electronic Data Capture (REDCap) tools hosted at the Medical University of Innsbruck [13]. REDCap is a secure Web-based application designed to support data capture for research studies, providing (1) an intuitive interface for validated data entry, (2) audit trails for tracking data manipulation and export procedures, (3) automated export procedures for seamless data downloads to common statistical packages, and (4) procedures for importing data from external sources. The link to the survey was available on the website of the NCS (https://www.curingcoma.org/home).

### Questionnaire Design

Survey questions can be found in Supplemental Table 3. The questions in Sect. 1 of the survey classified respondents on the basis of practice setting, (sub)specialty, years of clinical experience, and current practices for comatose patients. In Sect. 2 of the survey, respondents were asked to select cardinal features of coma that must be present to establish the diagnosis of coma from a predefined list (eight items) without weighting the importance of each feature. These cardinal features were selected by review of the literature and by expert consensus of the Prospective Studies Working Group members. On the basis of the respondents’ definitions of coma, branching logic was used to query for the top five most common etiologies of coma in their institution from a selection of 19 different possible etiologies.

Participants were then asked to grade their agreement on the following coma features, developed by consensus among the panel of experts, using a Likert scale (1 = “I fully agree” to 10 = “I fully disagree”) [14]: (1) no command following; (2) no intelligible speech or recognizable gesture; (3) no volitional movement (reflexive movement, such as extensor or flexor posturing, withdrawal from pain, and triple flexion, may occur); (4) no visual pursuit, fixation, saccade to stimuli, or eye opening or closing to command; (5) the above criteria are not due to use of paralytic agents, active use of sedatives, or another neurologic or psychiatric disorder (e.g., locked-in syndrome, neuromuscular disorder, catatonia, akinetic mute, abulia, conversion disorder); and (6) the patient does not have evidence of cognitive motor dissociation (i.e., the covert ability to follow commands) based on electrophysiological testing or functional imaging, if such testing is available (Table 1).

**Table 1:** Expert Consensus Definition of Coma provided in the survey

Coma is defined by the absence of sustained spontaneous or stimulus-induced arousal/wakefulness. All of the following criteria must be met on clinical examination to establish the diagnosis of coma:
1. No command-following, and
2. No intelligible speech or recognizable gesture, and
3. No volitional movement (reflexive movement such as extensor or flexor posturing, withdrawal from pain, triple flexion may occur), and
4. No visual pursuit, fixation, saccade to stimuli, or eye opening or closing to command, and
5. The above criteria are not due to use of paralytic agent, active use of sedatives, another neurologic or psychiatric disorder (e.g., locked-in syndrome, neuromuscular disorder, catatonia, akinetic mute, abulia, conversion disorder), and
6. The patient does not have evidence of cognitive motor dissociation (i.e. the covert ability to follow commands) based on electrophysiological or functional imaging, if such testing is available.

Additional questions focused on the duration of coma, diagnostic tools to evaluate comatose patients (25 predefined items), sedation practices, and detailed questions on the use and availability of electroencephalography (EEG) monitoring. In Sect. 3, respondents were instructed to indicate the top five most common etiologies of coma in their practice setting based on the provided definition of coma (18 items, excluding medically induced coma). Questions in Sect. 4 focused on management strategies of patients in coma (practices in neurological examination, pharmacological and nonpharmacological interventions to stimulate arousal, rehabilitation trajectory, discharge disposition). Section 5 focused on prognostication (top three most important elements used for prognostication in comatose patients, ten predefined items) and local policy for withdrawal of life-sustaining therapies (WLST). Finally, questions on future coma research areas and the willingness to participate in future studies were presented.

### Data Storage

Anonymized data were collected in a Web-based electronic case report form (REDCap) hosted at the Medical University of Innsbruck and stored in a secure database that was not accessible directly from the Internet.

### Statistical Analysis

After removal of duplicate responses, descriptive statistics were performed, and results are presented as frequencies and valid percentages. The denominator of each question referred to completed responses and therefore differed across questions. Fisher’s exact test was used to compare respondents who agreed or disagreed with the preestablished coma definition. The Fleiss *κ* statistic was used to express the level of interrater agreement on each of the eight predefined cardinal features of coma. According to Landis and Koch [15], the interpretation of the levels of agreement based on the *κ* values is as follows: < 0, poor; 0.01–0.20, slight; 0.21–0.40, fair; 0.41–0.60, moderate; 0.61–0.80, substantial; and 0.81–1.00, almost perfect. A *p* value < 0.05 was set as the statistically significant threshold. Statistics were performed with IBM SPSS Version 24.0 (IBM SPSS Statistics, Armonk, NY) and R version 4.0.2 with “irr” package version 0.84.1 and “rel” version 1.4.2.

## Results

A total of 258 health care workers from 41 countries completed the survey. Overall, complete survey responses (44 questions, five sections) were available in 84% of responses. By section, 99% completed Sect. 1 (identifying respondents), 93% completed Sect. 2 (defining and diagnosing coma), 89% completed Sect. 3 (etiology of coma), 89% completed Sect. 4 (management of patients in coma), and 89% completed Sect. 5 (attitudes toward prognosis). There was no difference in participant characteristics between those who completed diverse sections of the case report form and those who incompletely responded to each section.

### Participant Characteristics

**Fig. 1:**
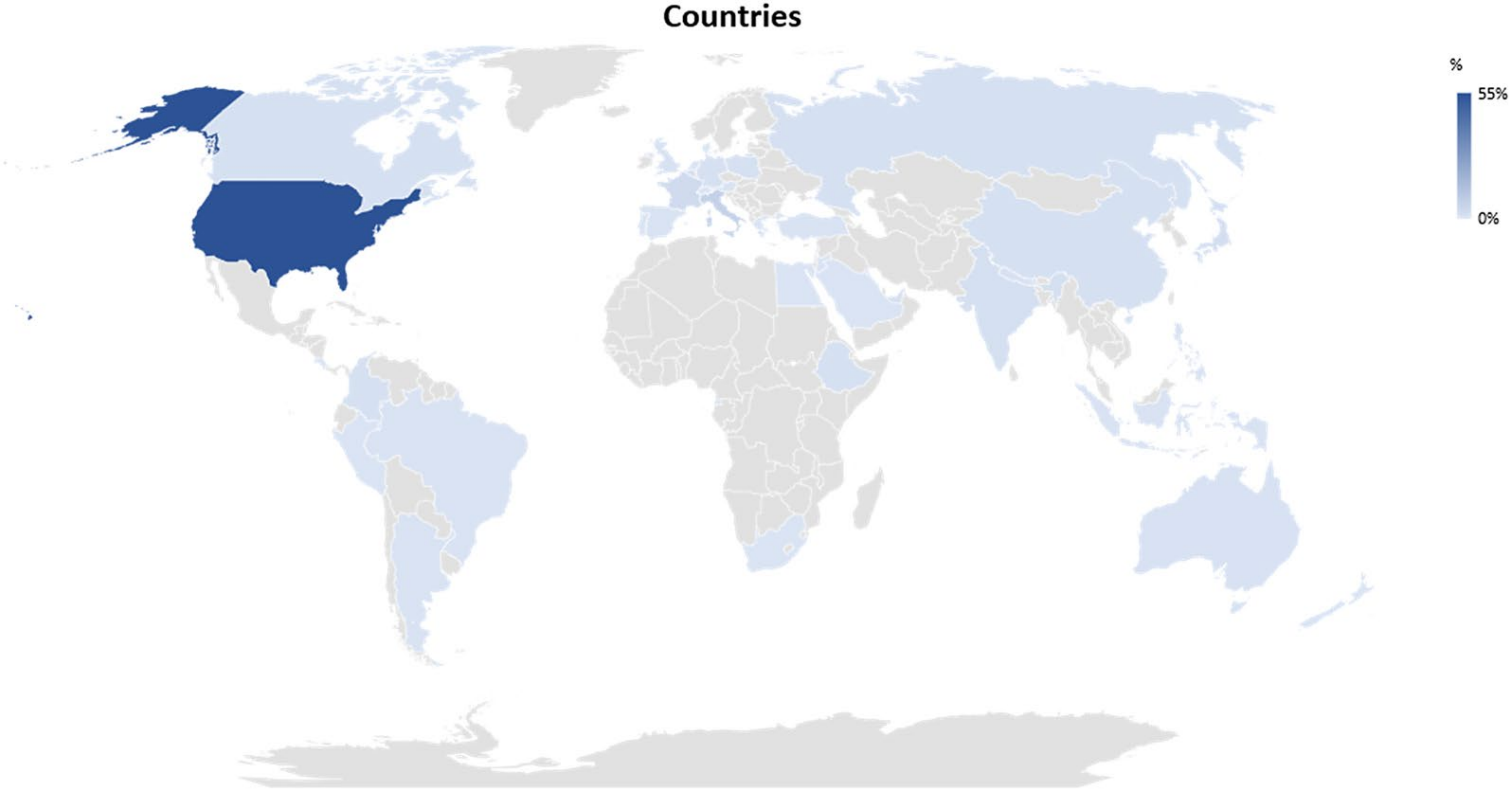
Countries of respondents contributing to the survey. The figure displays the number of respondents per country given in percentages

Most respondents were located in the United States (55%) followed by Europe (21%), Asia (17%), and Latin America (4%) (Fig. 1, Supplemental Table 4). The respondents represented a wide range of practice experience, from < 5 years (22%) to > 20 years (29%). The majority were from academic hospitals (*n* = 231 of 258; 90%), were physicians (*n* = 213 of 257; 83%), or were affiliated with neurology (*n* = 120 of 258; 47%), critical care (*n* = 65 of 258; 25%), neurosurgery (*n* = 20 of 258; 8%), or anesthesia (*n* = 15 of 258; 6%). Most respondents were trained in neurocritical care (*n* = 193 of 252; 77%), stroke (*n* = 46 of 252; 18%), or critical care medicine (*n* = 45 of 252; 18%). The majority of participants (78%) indicated that they treated on average > 15 adult comatose critically ill patients per month, whereas 17% treated between 1 and 15 patients per month, and 3% were not involved in acute critical care management. Only 31% of respondents treated pediatric patients with coma.

### Cardinal Features of Coma

Before respondents were provided with the expert panel’s definition for coma, the respondents were asked to select cardinal features that must be present to define coma out of a list of eight items (Table 2). We found marked variability in the selection and combination of these features, resulting in 89 different combinations and a median of four features selected per respondent (interquartile range 2–7). Only 15% of respondents (*n* = 37 of 252) selected all eight features of coma. When analyzing the agreement on individual features, we found substantial interrater agreement on absence of wakefulness (*κ* = 0.764), whereas moderate agreement (*κ* = 0.440–0.588) was found for five features (Glasgow Coma Score [GCS] ≤ 8; failure to respond to visual, verbal, or tactile stimulation; no command following; no eye-opening; no pursuit, fixation, or saccades to stimuli), and a fair agreement (*κ* = 0.383–0.394) was obtained for cognitive motor dissociation and no intelligible speech or gesture.

**Table 2:** Respondent selection of cardinal features of coma (*N* = 252 respondents)

	N, (%)	Fleiss *κ*^a^
Absence of wakefulness	204 (81)	0.764
Glasgow Coma Score ≤ 8	161 (64)	0.588
Failure to respond purposefully to visual, verbal or tactile stimuli based on clinical exam	152 (60)	0.552
Inability to follow commands (excluding aphasic patients)	146 (58)	0.529
No eye-opening	134 (53)	0.482
No visual pursuit of objects, fixation or saccade to stimuli	123 (49)	0.440
No evidence of cognitive motor dissociation (i.e. the covert ability to follow commands) based on exam, neurophysiological studies or functional imaging	111 (44)	0.394
No intelligible speech or recognizable gesture	108 (43)	0.383

Question in the survey: In your opinion, which of the following are considered cardinal features of coma (i.e., must be present to establish the diagnosis)? (Click all that apply.)

^a^Fleiss ĸ defines the level of agreement for each variable among respondents (< 0 poor agreement, 0.01–0.20 slight agreement, 0.21–0.40 fair agreement, 0.41–0.60 moderate agreement, 0.61–0.80 substantial agreement, 0.81–1.00 almost perfect agreement)

### Definition of Coma

**Fig. 2:**
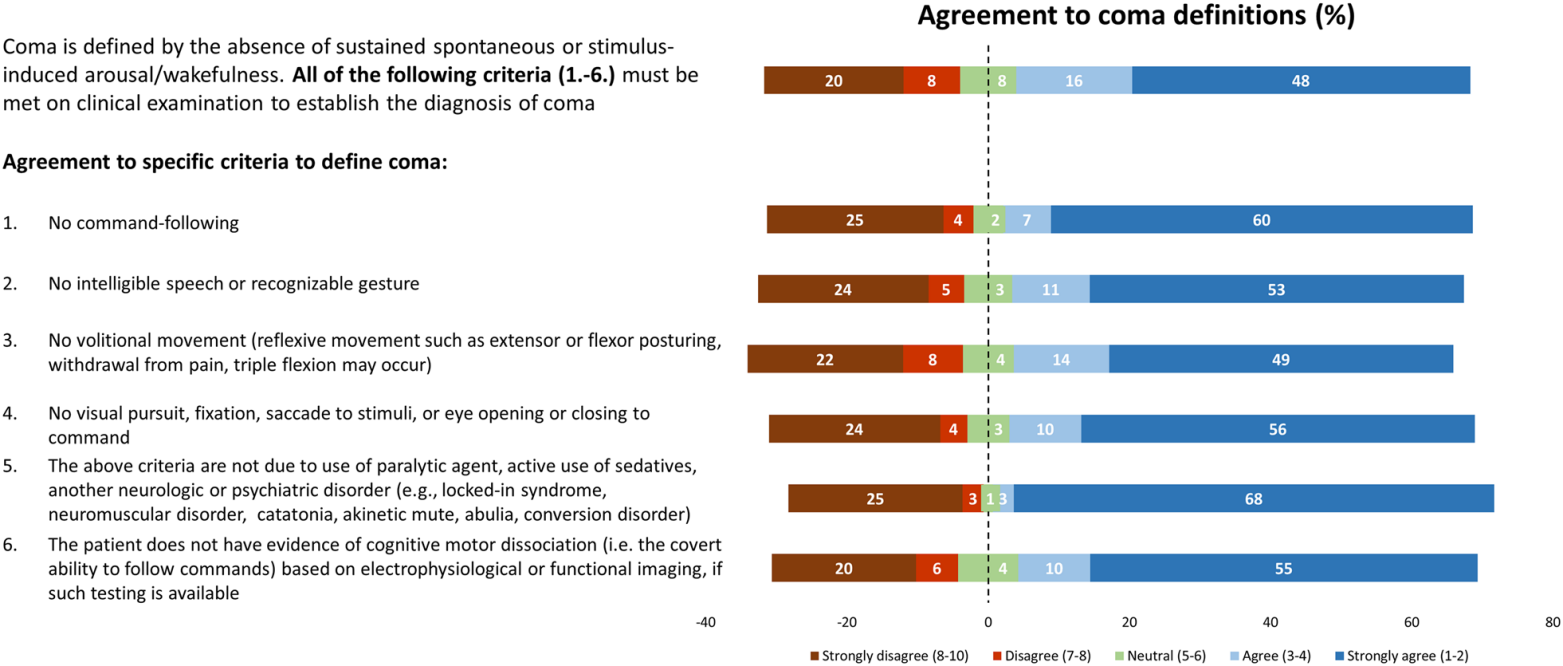
Agreement on the definition of coma (*n* = 238 respondents) based on Table 1. Bars reflect the percentage of agreement/disagreement for the overall definition of coma and each subfeature (1–6) provided in Table 1. Survey question: To what degree do you agree with the definition of coma as described above (1 = “I fully agree” to 10 = “I fully disagree”)?

For all subsequent questions, the survey referred to a definition of coma developed through consensus of the expert panel (Table 1). The overall level of agreement on this definition was 64% (*n* = 153 of 238), with 28% of respondents disagreeing (*n* = 67 of 238) and 8% neither agreeing nor disagreeing (*n* = 18 of 238). Details on response distribution are displayed in Fig. 2. Respondents who did not agree with the expert consensus definition did not differ from those who agreed in terms of years in practice, practice setting, (sub)speciality, or continent of origin (Supplemental Table 5). Participants strongly agreed that confounders (e.g., paralytic agent, active use of sedatives, other neurologic or psychiatric disorders) should be excluded. Regardless, 20–25% of respondents strongly disagreed on the features included in the expert definition of coma (Fig. 2).

### Coma Etiology and Diagnostic Approach

**Fig. 3:**
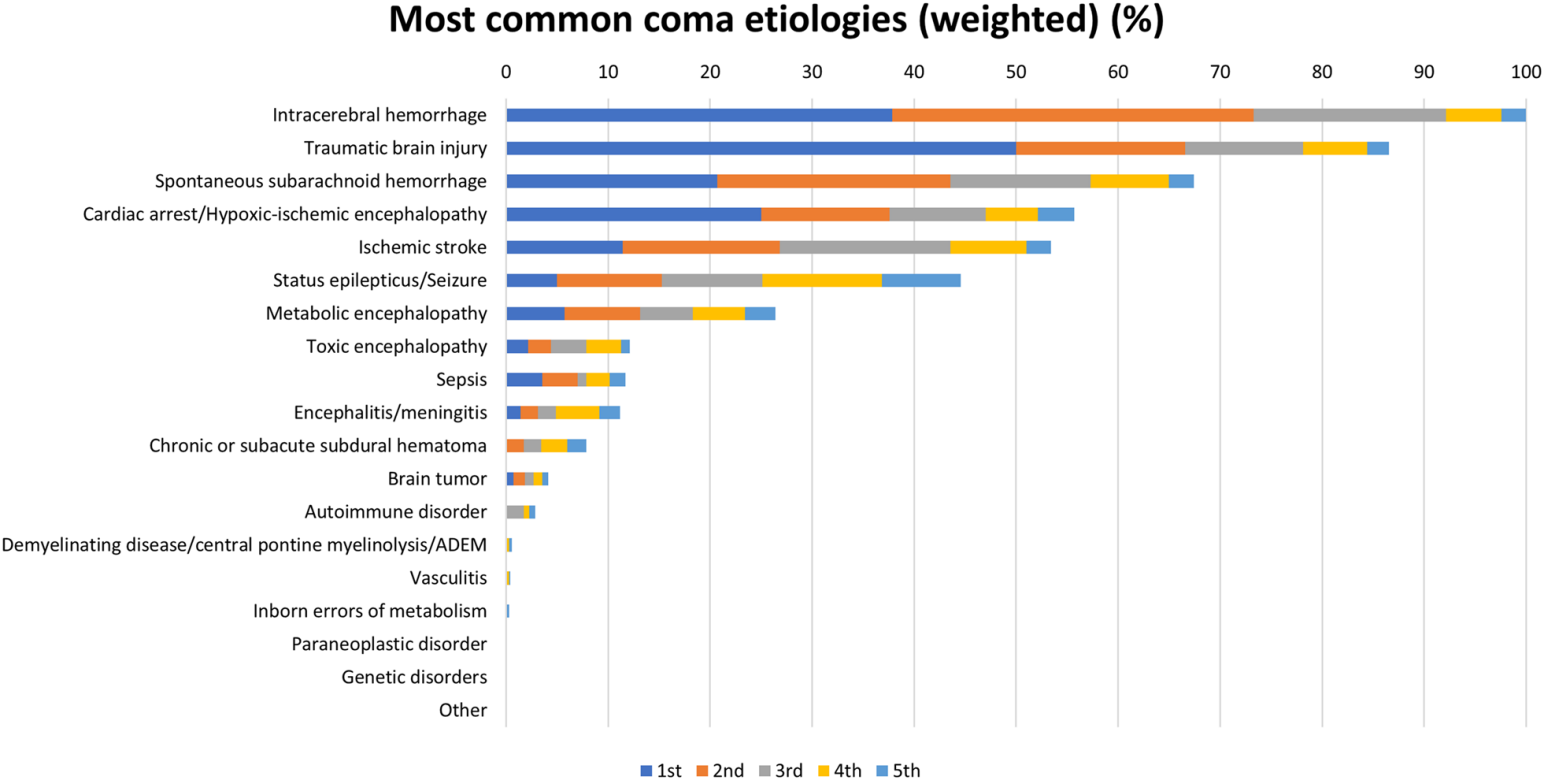
Most common etiologies of coma weighted by the five most common causes. Survey question: Rank the top five most common etiologies of coma that you encounter in your institution based on the definition of coma provided above. Bars represent the selection of etiologies based on the most common (blue), second most common (orange), third most common (gray), fourth most common (yellow), fifth most common (light blue) etiology of coma. Data are given in percentage and weighted based on the grading of respondents, normalized to the most common etiology (intracerebral hemorrhage). The answers were weighted based on the most common (multiplied by 5), the second most common (multiplied by 4), the third most common (multiplied by 3), the fourth most common (multiplied by 2) and the 5th most common etiology (multiplied by 1)

Most respondents reported treating between one and 15 patients per month with coma lasting for at least 24 h, whereas prolonged coma (lasting > 7 days) was uncommonly reported (Supplemental Fig. 1). Excluding patients with sedation-related coma (which represented the most common cause of coma by 24% of respondents; Supplemental Fig. 2), traumatic brain injury (TBI) was the most common etiology, whereas intracerebral hemorrhage (ICH) was indicated by all respondents as one of the five most common etiologies (Fig. 3). Uncommon causes of coma included infection, inflammatory disorders, genetic disorders, and tumors.

The majority of respondents (63%) indicated that coma can be diagnosed without a specific duration of cardinal features, whereas a duration of ≥ 24 h, ≥ 3 days, and ≥ 7 days was important for 12%, 3%, and 3% of respondents, respectively. The GCS was the most frequently used clinical assessment tool for comatose patients (*n* = 222 of 236; 94%) [16], followed by a complete neurological examination (*n* = 185 of 236; 78%) and the National Institutes of Health Stroke Scale (NIHSS; *n* = 115 of 236; 49%). In contrast, other clinical evaluation tools were infrequently used: the Confusion Assessment Method for the Intensive Care Unit (CAM-ICU; *n* = 68 of 236; 29%) [17], the Full Outline of Unresponsiveness (FOUR) score (*n* = 51 of 236; 22%) [18], and the Coma Recovery Scale–Revised (CRS-R) (*n* = 29 of 236; 12%) [19]. Two thirds of respondents (*n* = 154 of 235; 66%) reported regularly following a clinical protocol for sedation interruption in comatose patients when no specific contraindication existed (e.g., raised intracranial pressure), whereas 26% (*n* = 60 of 235) used sedation interruption sometimes, 4% used it rarely (*n* = 10 of 235), and 5% never used it (*n* = 11 of 235).

### Management of Patients in Coma

**Fig. 4:**
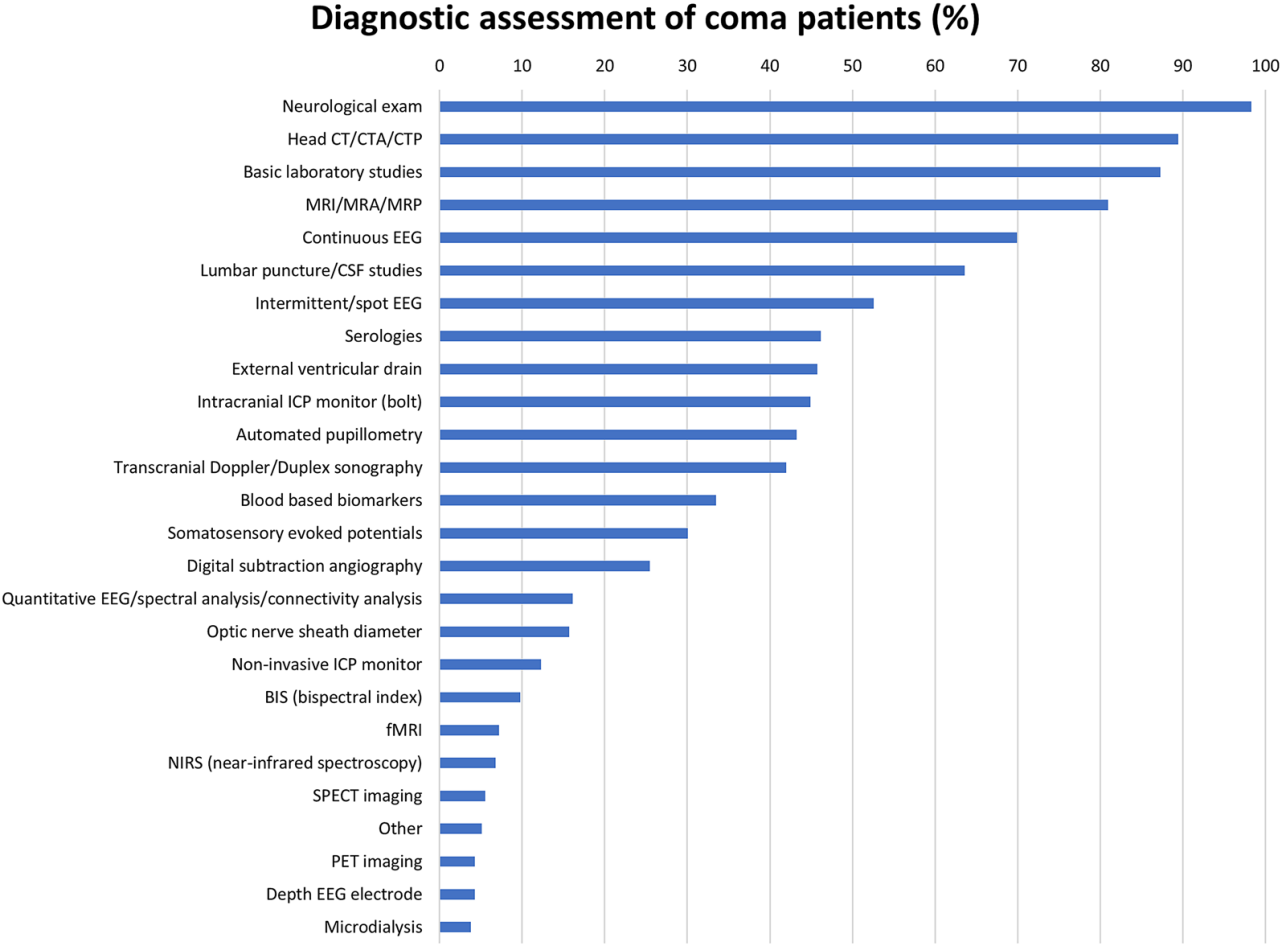
Diagnostic tools in the evaluation of comatose (≥ 24 h) patients (*n* = 236/258). Survey question: Which of the following tools do you routinely use in the diagnostic evaluation of these patients in coma (present ≥ 24 h)? CT, computed tomography; CTA, CT angiography; CTP, CT perfusion; MRI, magnetic resonance imaging; MRA, MR angiography; MRP, MR perfusion; EEG, electroencephalography; ICP, intracranial pressure; SPECT, single-photon emission computerized tomography; PET, positron emission tomography

Neurological examination was the most commonly used management tool for patients with prolonged coma (≥ 24 h; 98%), followed by EEG (either intermittent or continuous monitoring, 94%), and neuroimaging (head computed tomography, 89%; magnetic resonance imaging [MRI], 81%) (Fig. 4). Neurologic examination of comatose patients was commonly performed by neurointensivists (48%, *n* = 111 of 233) and at least once daily (96%, *n* = 223 of 233; Table 3). Regarding eye-opening, a key component of the GCS, 73% of respondents indicated that they regularly managed at least one patient per month with “eyes open coma” (eyes remain open despite the patient being unarousable and unresponsive) [20].

**Table 3:** Management of patients in coma

	N, (%)
Specialists performing neurological examination (N=233)	
Neurointensivist	111 (48)
Attending physician	53 (23)
Neurologist	21 (9)
Other	19 (8)
Advanced Practice Provider	15 (6)
Medical Intensivist	13 (6)
Surgical Intensivist	1 (1)
Frequency of routine neurologic examination (N = 233)	
Upon admission and every hour	8 (3)
Upon admission and every 2 h	8 (3)
Upon admission and every 4 h	17 (7)
Upon admission and every 8 h	27 (12)
Upon admission twice daily	71 (30)
Upon admission once daily	92 (39)
Other	10 (4)
Pharmacological interventions to stimulate arousal in patients with coma ≥ 24 h (N = 226)	
Sedation vacation	200 (88)
Electrolyte/endocrine correction	125 (55)
Amantadine	115 (51)
Osmotic therapy	112 (50)
Modafinil	83 (37)
Antidote for drug or illicit drug overdose	80 (35)
Sedation reversal	72 (32)
Steroids	68 (30)
Methylphenidate	67 (30)
Plasma exchange/plasmapheresis	51 (23)
Intravenous immunoglobulin	46 (20)
Amphetamine/dextroamphetamine	27 (12)
Levodopa	26 (12)
Zolpidem	17 (8)
Dopamine agonist	11 (5)
Other	10 (4)
Non-pharmacological interventions to stimulate arousal in patients with coma ≥ 24 h (N = 258)	
Sensory stimulation	76 (29)
Median nerve stimulation	13 (5)
Vagal nerve stimulation	12 (5)
Transcranial magnetic stimulation	5 (2)
Deep brain stimulation	8 (3)
Transcranial direct current stimulation	5 (2)
Other	18 (7)

More than half of respondents (*n* = 116 of 215; 54%) reported using standard EEG only. EEG services are, in principle, available 24/7 in the majority of institutions (*n* = 132 of 212; 62%), but 16% (*n* = 33 of 212) indicated limited hours per day, seven days a week, 15% (*n* = 31 of 212) had access only on weekdays, and 2% (*n* = 4 of 212) had access ≤ 5 days a week. Advanced neuroimaging techniques (e.g., functional MRI, positron emission tomography, single-photon emission computerized tomography) and invasive neuromonitoring techniques (e.g., depth electrodes, cerebral microdialysis) were uncommonly used for routine diagnosis and management of comatose patients (Fig. 4).

The most common pharmacologic interventions to stimulate arousal in patients with coma (≥ 24 h) were sedation vacation (*n* = 200 of 226; 88%) or sedation reversal (*n* = 72 of 226; 32%) and the use of neurostimulants (amantadine, *n* = 115 of 226 [51%]; modafinil, *n* = 83 of 226 [37%]; methylphenidate, *n* = 64 of 233 [28%]). Nonpharmacological interventions (vagal nerve stimulation, deep brain stimulation, transcranial magnetic stimulation) were rarely used. Only sensory stimulation was more commonly used (*n* = 76 of 258; 29%) (Table 3).

### Prognostication and Rehabilitation Trajectories in Comatose Patients

**Fig. 5:**
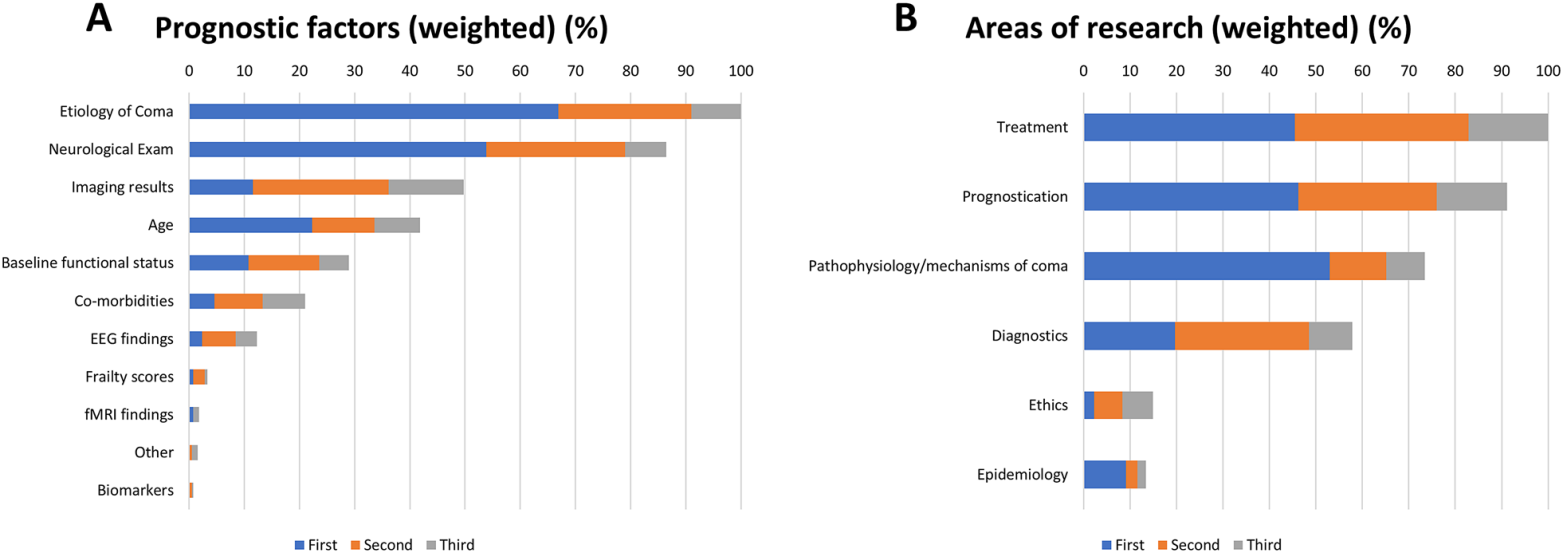
**a** Elements commonly used to prognosticate in coma patients (*n* = 226 of 258). Most important prognostic factors (first) were the etiology of coma (*n* = 87 of 226, 38%), findings in neurological examination (*n* = 70 of 226, 31%) and age (*n* = 29 of 226, 13%). The top three most important factors were etiology of coma (*n* = 170 of 226, 75%), findings in neurological examination (*n* = 149 of 226, 66%) and neuroimaging (*n* = 115 of 226, 51%). Bars represent the cumulative incidence for ranking the top three elements used for prognostication normalized to “etiology of coma” (100%). Survey question: Please rank the top three (first, second, third) most important elements you utilize for prognostication in comatose patients. The answers were weighted based on the most common (multiplied by 3), the second most common (multiplied by 2), the third most common (multiplied by 1). **b** Areas of coma research for coma patients. Survey question: What areas of coma research focus do you feel are most important/urgent? The answers were weighted based on the most common (multiplied by 3), the second most common (multiplied by 2), the third most common (multiplied by 1)

Respondents reported that the following determinants contributed to prognostication: etiology of coma (75%), neurological examination (66%), neuroimaging findings (51%), and age (36%) (Fig. 5a). The majority of respondents indicated available rehabilitation services and units for the transfer of patients (*n* = 106 of 230; 46%); however, 43% (*n* = 99 of 230) did not officially partner with rehabilitation facilities, which led to transfer of patients to a variety of different centers and levels of care. The most common discharge dispositions for comatose patients who survived hospitalization were long-term acute care hospitals (*n* = 84 of 230; 37%), followed by skilled nursing facilities (*n* = 41 of 230; 18%) and acute rehabilitation facilities (*n* = 41 of 230; 18%). Fewer than 10% of patients with coma were reported to be discharged home with assistive services (Supplemental Fig. 3).

Twenty-nine percent (*n* = 65 of 227) of respondents reported having a formal policy or protocol for WLST among patients in coma. Overall, 43% (*n* = 97 of 226) of respondents felt that no fixed observation period was necessary prior to WLST among comatose patients, whereas 14% (*n* = 31 of 226) provided fixed time windows, and 43% (*n* = 98 of 226) indicated that the approach varied according to the cause of coma. Ethicists were regularly involved in the discussions about WLST in patients with coma according to 6% (*n* = 13 of 225) of respondents, whereas 38% of respondents (*n* = 86 of 225) indicated that an ethicist was occasionally involved in WLST decisions.

### Future Research

The majority of respondents indicated that future research for coma patients should focus on treatment (*n* = 202 of 232; 87%), prognostication (*n* = 180 of 232; 78%), pathophysiology (*n* = 127 of 232; 55%), and diagnostics (*n* = 120 of 232; 52%) (Fig. 5b). In addition, distinguishing coma from unresponsive wakefulness state was considered a key factor for clinical care and research by 89% (*n* = 213 of 239) of respondents, and 30% (*n* = 70 of 232) indicated that pathophysiology and mechanisms of coma as highest priority research areas.

Although few respondents (13%) routinely followed coma patients longitudinally after ICU discharge, the majority indicated that follow-up of comatose patients would be feasible by telephone interview (65–73%) or video (33–37%) and, to a lesser extent, as in-person follow-up (20–25%).

Most respondents were willing to participate in future studies: 90% were interested in an annual survey regarding coma, and 82% indicated an interest in participating in prospective observational trials of comatose patients.

## Discussion

This study represents the first online survey initiated by the NCS CCC [21] and is the first, to our knowledge, to explore global attitudes regarding the definition of acute coma, common etiologies, management practices, and approaches to prognostication in clinical practice.

Overall, we identified substantial discrepancies in opinions regarding the definition of coma. Fewer than two thirds of respondents agreed with the constellation of clinical features proposed by the expert panel to define coma. This highlights the need for education even in academic centers with specialists having long-year experience in the care of patients with coma. Considering each feature separately, 64% of respondents felt that a GCS score ≤ 8 (which is often used to define coma for research and clinical purposes) is a necessary component of the diagnosis. Although a GCS ≤ 8 has been considered a hallmark feature of coma, several limitations of the GCS have been identified, notably incomplete assessment of intubated patients, lack of items that distinguish coma from other DoC, failure to address brainstem reflexes, and limited ability to differentiate prognosis among patients with the lowest GCS [18, 22, 23]. Indeed, it is possible to have a GCS ≤ 8 in patients who are able to follow verbal commands or are localizing to pain and would not otherwise be considered comatose by most practitioners (e.g., eyes 2, motor 5, verbal 1 or eyes 1, motor 6, verbal 1) [24]. Additionally, the eye opening component may be misleading in coma, particularly because 73% of survey responders acknowledged treating at least one patient with eyes open coma [20] per month. It remains possible that respondents refer to an “eyes open state” in VS/UWS and not in coma because eyes open coma is clinically indistinguishable from VS/UWS, which has historically been viewed as a distinct condition that can persist for decades. Limitations in the GCS led to the development of the FOUR score [18], although this scoring system also does not capture the phenomenon of eyes open coma and does not distinguish between different DoC diagnoses. Because the GCS and FOUR score both positively weight spontaneous eye opening, the use of these scores for prognostication may generate overly optimistic outcome estimations for patients with eyes open coma. Conversely, lack of assessment of behaviors that suggest emergence from coma to a minimally conscious state (e.g., automatic motor responses, visual fixation) may generate overly pessimistic outcome estimations, whereas evidence of MCS is consistent with a more favorable prognosis [25].

Although the CRS-R [26] is considered the gold standard for assessment of DoC in the subacute and chronic setting [27, 28], it was rarely used in the acute setting in this survey (12%), possibly because it takes between 15 and 30 min to complete. Abbreviated versions of the CRS-R have been validated [29, 30], and the reliability and validity of a streamlined version of the CRS-R known as the CRSR-FAST (CRS-R For Accelerated Standardized Testing), which aims to shorten administration time for patients in the ICU to less than 8 min, is currently under investigation (ClinicalTrials.gov identifier NCT03549572). Because few respondents reported caring for patients with coma in the postacute and chronic care settings, reports of scales routinely used for assessments are skewed toward those commonly applied in the ICU setting.

Interestingly, respondents preferred the use of scales (e.g., the GCS) to features of the neurological examination in the assessment of comatose patients. It should be emphasized that both the GCS and the FOUR scores were designed to grade the level of consciousness and not to define coma. In the same line, the National Institutes of Health Stroke Scale and Confusion Assessment Method for the Intensive Care Unit are approved for stroke and delirious patients, respectively, and not to grade the level or unresponsiveness.

After excluding patients with medically induced coma, TBI was the leading etiology reported, although ICH was more common when accounting for all top five ranked etiologies. It is worth noting that the majority of survey responders were from US academic centers, which are often tertiary or quaternary care sites and not consistently level 1 trauma centers. This factor may bias against TBI as a common etiology. Indeed, according to the Centers for Disease Control and Prevention, there were approximately 288,000 TBI-related hospitalizations in 2014 in the United States [31], compared to approximately 40,000–67,000 ICHs per year [32]. Similarly, spontaneous subarachnoid hemorrhage was ranked third cumulatively as a cause of coma, despite having an incidence worldwide of 6.1 per 100,000 patient-years, which is equivalent to ~ 20,000 new cases per year in the United States [33]. Conversely, cardiac arrest was the fourth leading cause of coma in this survey, despite an incidence of more than 350,000 out-of-hospital cardiac arrests per year and 200,000 in-hospital cardiac arrests per year [34], with an estimated 55,000–165,000 survivors annually. It is possible that post cardiac arrest, patients are infrequently managed by neurointensivists (who were the most common survey respondents), leading to underreporting of this clinical condition. Because of the obvious biases in reporting coma etiologies, the CCC plans to launch a global incidence study to more precisely assess the incidence and leading causes of coma.

We found that common diagnostic approaches to coma included the neurological examination, EEG monitoring, and basic neuroimaging, such as computed tomography or MRI. The high rate of EEG availability (24/7, 62%) and the use of continuous EEG monitoring (69%) should be interpreted in the context of respondents from academic centers and may not represent standard approaches in nonacademic institutions. Still, fewer than 10% of respondents indicated the use of advanced diagnostic techniques, such as positron emission tomography, single-photon emission computerized tomography, or functional MRI. The lack of access to these diagnostic tools in clinical settings is notable because part of the suggested definition of coma devised by the expert panel includes absence of cognitive motor dissociation, which is typically diagnosed with either functional imaging or quantitative EEG analyses [6, 35–37]. Although functional neuroimaging may be logistically challenging for patients who may not be medically stable for transport and may not be economically or technically viable at most sites, conversely EEG techniques for assessing covert consciousness may be more broadly available. Indeed, the source code for EEG evaluation of cognitive motor dissociation has been made publicly available [6] and could conceivably among others be scaled to integrate with current EEG monitoring systems.

Interruption of sedation was the most common pharmacological intervention, yet only 66% of respondents reported regularly stopping sedation when no specific contraindication existed (e.g., ventilator dyssynchrony, elevated ICP). Regarding the treatment of patients in coma, our data suggest that very little progress has been made over the last decades. Amantadine was routinely used by 51% of responders, whereas modafinil was used by 37%. The data supporting amantadine as a neurostimulant, including two randomized controlled trials of patients with severe TBI [38, 39], is substantially more robust than data supporting modafinil. A recent study in ICU patients with ischemic or hemorrhagic stroke suggested that more than half of patients have some improved arousal with amantadine in the acute ICU setting, whereas no patient responded to modafinil [40]. The popularity of modafinil despite a dearth of supportive data suggests that additional studies and education are needed regarding the use of neurostimulants, particularly for coma.

Finally, the most important factors for prognostication according to respondents were the etiology of coma, the neurological examination, imaging results, and age (Fig. 5a). Notably, 43% of respondents indicated that there was no minimum time needed to assess patients prior to WLST after the diagnosis of coma was established, whereas an equal number felt that the waiting time prior to withdrawal should vary by coma etiology. The lack of empirical data likely contributes to early decisions to WLST after TBI (median time from injury to WLST was 3 days) [41, 42]. Given the inherent uncertainty surrounding prognostication, further exploration of attitudes toward WLST among comatose patients seems warranted.

There are strengths and limitations to our study. The major strength is the provision, for the first time, of a picture of the diagnosis and treatment of comatose patients in clinical practice, comparing various countries and populations at risk. In the absence of practice guidelines for the assessment of coma independent of the underlying etiology, a clear understanding of the variability of the diagnostic and treatment attitudes and practices provides the background for a systematic review of the literature and for developing evidence-based diagnostic and therapeutic recommendations. In light of this, certain limitations must also be addressed. Most respondents were from US academic centers and there was limited representation from developing nations. As noted previously, the referral structure of tertiary academic centers and trauma centers in the United States may skew data regarding etiologies of coma. Additionally, recall bias limits interpretation of the frequency of medical interventions and discharge dispositions. In addition, we did not successfully reach out to health care clinicians involved in the subacute and chronic care for patients with DoC. Most respondents were intensivists and few had longitudinal follow-up of coma patients in the subacute or chronic setting. This may have influenced the information provided beyond the acute ICU care, such as pharmacologic interventions. Moreover, limited clinical experience with long-term outcomes among comatose patients may bias attitudes toward prognostication and WLST. In addition, only few respondents cared for pediatric patients, in whom coma etiologies and treatment likely vary substantially from adults. Last, we did not reach consensus among health care providers on the proposed definition of coma. It is also conceivable that some respondents who strongly disagreed with certain components had a misunderstanding of the rating scale. Moreover, we did not include control questions to assess validity of certain answers. However, on review of individual respondent-level data, we were unable to detect clear trends that would suggest systematic error.

## Conclusions

Coma definition and management strategies vary among health care clinicians. This provides the rationale for planning and developing diagnostic and therapeutic guidelines for evidence-based management of comatose patients. On the basis of the highest agreement for absence of wakefulness as one cardinal feature of coma, more emphasis should be placed on objectively measuring wakefulness. Because the diagnostic and therapeutic techniques employed to manage comatose patients do not seem to have improved substantially in recent years, targeted research aimed at disaggregating coma endophenotypes and advancing novel therapeutic interventions is urgently needed. Furthermore, longitudinal care of comatose patients and those with DoC years after brain injury is urgently needed to understand clinical trajectories of individual patients.

## Supplementary Information

Below is the link to the electronic supplementary material.

Supplementary file1 (DOCX 70 kb)

## Abbreviations

CAM-ICU: Confusion Assessment Method for Intensive Care Unit
CCC: Curing Coma Campaign
DoC: Disorders of consciousness
FOUR: Full Outline of UnResponsiveness score
GCS: Glasgow Coma Scale
ICH: Intracerebral hemorrhage
NCS: Neurocritical Care Society
NIHSS: National Institutes of Health Stroke Scale
PET: Positron-emission-tomography
SPECT: Single-photon emission computerized tomography
TBI: Traumatic brain injury
WLST: Withdrawal of Life-Sustaining Treatment
