# Supplementary material for: The Curing Coma Campaign International Survey on Coma Epidemiology, Evaluation, and Therapy (COME TOGETHER)
Source: Neurocrit Care. 2022 Feb 9;37(1):47–59. doi: 10.1007/s12028-021-01425-8 (PMC9283177; doi:10.1007/s12028-021-01425-8)
Supplement: Supplementary file 1 — Supplementary file1 (DOCX 70 kb) [file 12028_2021_1425_MOESM1_ESM.docx]

**Supplemental material**

**Supplemental Table 1**

The Curing Coma Campaign and its contributing members

| **First Name** | **Last Name** | **Email Address** |
| --- | --- | --- |
| Yama | Akbari | yakbari@gmail.com |
| Thomas | Bleck | tbleck@gmail.com |
| Melanie | Boly | boly@neurology.wisc.edu |
| Jan | Claassen | jc1439@columbia.edu |
| Neha | Dangayach | neha.dangayach@mountsinai.org |
| Michael | Diringer | diringerm@wustl.edu |
| Brian L | Edlow | bedlow@mgh.harvard.edu |
| Joseph | Fins | jjfins@med.cornell.edu |
| Brandon | Foreman | foremabo@ucmail.uc.edu |
| Emily | Gilmore | emily.gilmore@yale.edu |
| Olivia | Gosseries | ogosseries@uliege.be |
| Flora | Hammond | flora.hammond@rhin.com |
| Dan | Hanley | dhanley@jhmi.edu |
| Claude | Hemphill III | claude.hemphill@ucsf.edu |
| Theresa | Human | doctheresa.human6@gmail.com |
| Lori | Kennedy Madden | lkmadden@ucdavis.edu |
| Keri | Kim | [skim42@uic.edu](mailto:skim42@uic.edu) |
| Ariane | Lewis | [ariane.kansas.lewis@gmail.com](mailto:ariane.kansas.lewis@gmail.com) |
| Shraddha | Mainali | [shraddha.mainali@vcuhealth.org](mailto:shraddha.mainali@vcuhealth.org) |
| Geert | Meyfroidt | geert.meyfroidt@uzleuven.be |
| Martin | Monti | MMonti@mednet.ucla.edu |
| Naomi | Niznick | Naniznick@toh.ca |
| Paul | Nyquist | pnyquis1@jhmi.edu |
| Daiwai | Olson | daiwai.olson@utsouthwestern.edu |
| Adrian | Owen | uwocerc@uwo.ca |
| Soojin | Park | spark@columbia.edu |
| Nader | Pouratian | nader.pouratian@utsouthwestern.edu |
| Len | Polizzotto | Len_polizzotto@yahoo.com |
| Javier | Provencio | jp3b@virginia.edu |
| Louis | Puybasset | louis.puybasset@psl.aphp.fr |
| Risa | Richardson | Risa.Richardson@va.gov |
| Benjamin | Rohaut | benjamin.rohaut@sorbonne-universite.fr |
| Eric | Rosenthal | erosenthal@mgh.harvard.edu |
| Gisele | Sampaio-Silva | giselesampaio@hotmail.com |
| Aarti | Sarwal | asarwal@wakehealth.edu |
| Lori | Shutter | shutterla@upmc.edu |
| Jose | Suarez | jsuarez5@jhmi.edu |
| Amy | Wagner | wagnerak@upmc.edu |
| John | Whyte | jwhyte@einstein.edu |
| Briana | Witherspoon | Briana.Witherspoon@UTSouthwestern.edu |
| Wendy | Ziai | weziai@jhmi.edu |

**Supplemental Table 2**

Members of the *Prospective Studies Working Group* of the Curing Coma Campaign who designed the COME TOGETHER survey.

| Raimund Helbok, Verena Rass | Department of Neurology, Neuro-Intensive Care Unit, Medical University of Innsbruck, Austria |
| --- | --- |
| Ettore Beghi | Department of Neuroscience, Istituto di Ricerche Farmacologiche Mario Negri IRCCS, Milan, Italy. |
| Giuseppe Citerio | NeuroIntensive Care, ASST di Monza, Monza, Italy; School of Medicine and Surgery, Università Milano Bicocca, Milan, Italy |
| Joseph Giacino | Department of Physical Medicine and Rehabilitation, Spaulding Rehabilitation Hospital, Harvard Medical School, Boston, MA, USA |
| Daniel Kondziella | Department of Neurology, Rigshospitalet, Copenhagen University Hospital, and Faculty of Health and Medical Sciences, University of Copenhagen, Copenhagen, Denmark |
| Stephan Mayer | Department of Neurology, New York Medical College, Valhalla, NY, USA |
| David Menon | Division of Anaesthesia, University of Cambridge, Cambridge, UK |
| Tarek Sharshar | Neuro-anesthesiology and Intensive Care Medicine, Sainte-Anne Hospital, Paris-Descartes University, Paris, France and Experimental Neuropathology, Infection and Epidemiology Department, Institut Pasteur, Paris, France |
| Robert D Stevens | Departments of Anesthesiology and Critical Care Medicine, Neurology, and Neurosurgery, The Johns Hopkins University School of Medicine, Baltimore, MD, USA |
| Chethan P Venkatasubba Rao | Division of Vascular Neurology and Neurocritical Care, Baylor College of Medicine and CHI Baylor St Luke's Medical Center, Houston, TX, USA. |
| Paul Vespa | Departments of Neurology and Neurosurgery, David Geffen School of Medicine at UCLA, Los Angeles, CA, USA |
| Molly McNett | College of Nursing, The Ohio State University, Columbus, OH, USA |
| Jennifer Frontera | Department of Neurology, NYU Langone Health, New York, New York. |

**Supplemental Table 3**

Survey questions of the COMa EpidemioloGy, Evaluation and THERapy” (COMEtoGETHER) study

**Case Record Form**

SECTION 1: IDENTIFY RESPONDANT

SECTION 2: DEFINING AND DIAGNOSING COMA

SECTION 3: ETIOLOGY OF COMA

SECTION 4: MANAGEMENT OF PATIENTS IN COMA

SECTION 5: ATTITUDES TOWARD PROGNOSIS

| Contact Information |  |
| --- | --- |
| *Variable* | *Response Option* |
| Name | Free text |
| Title | Free text |
| Affiliation/Institution | Free text |
| Section/Department | Free text |
| Mailing Address | Free text |
| City, Country | Free text |
| Primary Email Contact | Free text |
| Secondary Email Contact | Free text |
| Primary Phone Contact | Free text |
| Secondary Phone Contact | Free text |
| Would you be interested in participating in an international survey about current practices in your institution for managing patients in coma? | No  Yes  (if yes, proceed with completion of questions below on page 2) |
| Would you be interested in participating in an international point prevalence study, where data on all coma patients in your unit is recorded for a set period of time? | No  Yes  If no, please indicate reason: |
| Are you able to obtain necessary Institutional Review Board (IRB) or Declaration of Helsinki approvals within a 3-month timeframe for data collection if required for the point prevalence study? | No  Yes |
| Does your institution allow for use of a central IRB? | No  Yes  Don’t know |
| What Electronic Medical Record does your hospital use? | Epic  Serna  Centricity  Other |

| SECTION 1: IDENTIFY RESPONDANT |  |
| --- | --- |
| Number of years in practice | 0-5, 6-10, 11-15, 16-20, 21-30, >30 |
| How would you describe the area in which you practice? | urban, suburban or rural |
| How would you describe your practice setting? | Academic/Teaching hospital  Non-teaching hospital  Public non-academic hospital  Private non-academic hospital  District or Government Hospital  Other (specify) |
| What type of practitioner are you? | Physician, NP, PA, nurse, pharmacist, other (specify) |
| What is your primary department affiliation? | Neurology, Neurosurgery, Surgery, Anesthesia, Critical Care, Psychiatry, Physical Medicine and Rehabilitation, Pediatrics, Internal medicine, Emergency medicine, Nursing, Pharmacy, Other (specify) |
| What is your subspecialty? (check all that apply) | Neurocritical Care, endovascular/interventional, vascular neurosurgery, stroke, general neurology, general neurosurgery, trauma surgery, critical care medicine, surgical critical care, cardiac/cardiothoracic surgery critical care, pulmonary critical care, anesthesia/critical care, Physical Medicine and Rehabilitation, other (specify) |
| How many adult (aged≥18 years) critically ill patients do you treat per month on average? | 0, 1-5, 6-10, 11-15, 16-30, >30 |
| What is the typical nurse to patient ratio for mechanically ventilated patients in your institution? | 1:1  1:2  1:3  1:4  Other (specify) |
| On average, how many *pediatric* patients (aged<18 years) do you treat per month? | 0, 1-5, 6-10, 11-15, 16-30, >30 |
| Do you currently follow your comatose patients after discharge?  *Indicate (yes/no/not sure) for each of the following:* | 1. By telephone at 3 months 2. By telephone at 6 months 3. By telephone at 12 months 4. In person at 3 months 5. In person at 6 months 6. In person at 12 months 7. By video at 3 months 8. By video at 6 months 9. By video at 12 months |

| SECTION 2: DEFINING AND DIAGNOSING COMA | |
| --- | --- |
| In your opinion, which of the following are considered cardinal features of coma (i.e., must be present to establish the diagnosis)? (click all that apply) | 1. Glasgow Coma Score ≤8 2. Absence of wakefulness 3. No eye-opening 4. No visual pursuit of objects, fixation or saccade to stimuli 5. Inability to follow commands (excluding aphasic patients) 6. No intelligible speech or recognizable gesture 7. Failure to respond purposefully to visual, verbal or tactile stimuli based on clinical exam 8. No evidence of cognitive motor dissociation (i.e. the covert ability to follow commands) based on exam, neurophysiological studies or functional imaging 9. Other(specify) |
| Please provide the BEST DEFINITION OF COMA used in your institution | Text |
| What duration of impaired consciousness should be present to meet your definition of coma? | No specific duration  ≥ 1 hour  ≥6 hours  ≥24 hours  ≥3 days  ≥7 days  other(specify) |
| On average, how many coma patients do you treat in the ICU setting per month? Specifically, patients in coma (based on your definition of coma) *for ≥1 hour?* | 0, 1-5, 6-10, 11-15, 16-30, >30 |
| On average, how many coma patients do you treat in the ICU setting per month? Specifically, patients in coma (based on your definition of coma) *for ≥24 hours?* | 0, 1-5, 6-10, 11-15, 16-30, >30 |
| On average, how many coma patients do you treat in the ICU setting per month? Specifically, patients in coma (based on your definition of coma) *for ≥7days?* | 0, 1-5, 6-10, 11-15, 16-30, >30 |
| On average, how many patients with unresponsive wakefulness syndrome or vegetative state do you treat in the ICU setting per month (defined as a condition of wakefulness without clinical signs of awareness. Such patients may open their eyes but exhibit only reflex [i.e. non-intentional] behaviors and are therefore considered unaware of themselves and their surroundings)? | 0, 1-5, 6-10, 11-15, 16-30, >30 |
| Rank the top 5 most common etiologies of coma that you encounter in your institution based on your definition of coma?  (Rank 1= most common... 5=fifth most common) | Medically induced coma  Traumatic brain injury  Ischemic stroke  Intracerebral hemorrhage  Spontaneous subarachnoid hemorrhage  Chronic or subacute subdural hematoma  Status epilepticus/Seizure  Encephalitis/meningitis  Demyelinating disease/central pontine myelinolysis/ADEM  Paraneoplastic disorder  Autoimmune disorder  Vasculitis  Brain tumor  Cardiac arrest/Hypoxic-ischemic encephalopathy  Sepsis  Metabolic encephalopathy (related to electrolyte abnormality, thyroid, adrenal, liver and/or renal dysfunction, or vitamin deficiency)  Toxic encephalopathy (illicit or iatrogenic drug overdose, poisoning)  Inborn errors of metabolism  Genetic disorders  Other (specify) |

| Please use the following strict definition of “coma” when responding to the questions below in sections 2 through 5.  *Coma is defined by the absence of sustained spontaneous or stimulus-induced arousal/wakefulness. All of the following criteria must be met on clinical examination to establish the diagnosis of coma:*  *1. No command-following; and*  *2. No intelligible speech or recognizable gesture; and*  *3. No movement (reflexive movement such as extensor or flexor posturing, withdrawal from pain, triple flexion may occur); and*  *4. No visual pursuit, fixation, saccade to stimuli, or eye opening or closing to command; and*  *5. The above criteria are not due to use of paralytic agent, active use of sedatives, another neurologic or psychiatric disorder (e.g., locked-in syndrome, neuromuscular disorder, catatonia, akinetic mute, abulia, conversion disorder).*  *6. The patient does not have evidence of cognitive motor dissociation (i.e. the covert ability to follow commands) based on electrophysiological or functional imaging, if such testing is available.* | |
| --- | --- |
| To what degree do you agree with the definition of coma as described above?  Scale of 1-10 from “I fully agree” to “I fully disagree” | **Scale of 1-10** |
| What duration of impaired consciousness should be present to meet the definition of coma? | No specific duration  ≥ 1 hour  ≥6 hours  ≥24 hours  ≥3 days  ≥7 days  other(specify) |
| What clinical assessment tools does your institution routinely use for patients in coma? (check all that apply) | Glasgow Coma Scale  Full Outline of UnReponsiveness Score (FOUR-score)  NIHSS  CRS-R  CAM-ICU  Full neurological exam  Other (specify) |
| Is it critical to distinguish between coma and unresponsive wakefulness state (or vegetative state) for the purposes of clinical care or research? | Yes  No  Don’t know |
| On average, how many patients with “eyes open coma” do you treat in the ICU setting per month (defined as above, but eyes are open and/or the patient is unable to close their eyes)? | 0, 1-5, 6-10, 11-15, 16-30, >30 |
| Do you follow a protocol for stopping sedation in all patients without a contraindication for stopping sedation (raised ICP, etc) at least once daily? | Always  Sometimes  Rarely  Never |
| How often do you use the following tools in the DIAGNOSTIC evaluation of these patients in coma (present ≥24 h)?  Check all that you routinely use as part of your diagnostic evaluation. | Neurological Exam  Basic laboratory studies  Lumbar puncture/CSF studies  Serologies  Blood based biomarkers  Somatosensory evoked potentials  Automated pupillometry  NIRS (near-infrared spectroscopy)  BIS (bispectral index)  Head CT/CTA/CTP  MRI/MRA/MRP  Digital subtraction angiography  PET imaging  SPECT imaging  fMRI  Transcranial Doppler/Duplex sonography  Continuous EEG  Intermittent/spot EEG  Quantitative EEG/spectral analysis/connectivity analysis  Optic nerve sheath diameter  Microdialysis  Depth EEG electrode  External ventricular drain  Intracranial ICP monitor (bolt)  Non-invasive ICP monitor  Other (specify) |
| IF YOU USE EEG:  How is EEG interpretation performed?  What is availability of EEG services?  What EEG metrics are used for assessing coma recovery? | Clinical review  Quantitative/connectivity analysis  Both  7 days a week, 24 hours a day  7 days a week, limited hours  Only on weekdays, 5 days a week, 24 hours a day  Only on weekdays, 5 days a week, but with limited hours  Less than 5 days a week  Continuous EEG  Intermittent EEG  Baseline EEG  Reactivity  EEG interpretation using clinical review  EEG interpretation using quantitative or connectivity analysis |

| SECTION 3: ETIOLOGY OF COMA | |
| --- | --- |
| Rank the top 5 most common etiologies of coma that you encounter in your institution based on the definition of coma provided above.  (Rank 1= most common... 5=fifth most common | Traumatic brain injury  Ischemic stroke  Intracerebral hemorrhage  Spontaneous subarachnoid hemorrhage  Chronic or subacute subdural hematoma  Status epilepticus/Seizure  Encephalitis/meningitis  Demyelinating disease/central pontine myelinolysis/ADEM  Paraneoplastic disorder  Autoimmune disorder  Vasculitis  Brain tumor  Cardiac arrest/Hypoxic-ischemic encephalopathy  Sepsis  Metabolic encephalopathy (related to electrolyte abnormality, thyroid, adrenal, liver and/or renal dysfunction, or vitamin deficiency)  Toxic encephalopathy (illicit or iatrogenic drug overdose, poisoning)  Inborn errors of metabolism  Genetic disorders  Other (specify) |

| SECTION 4: MANAGEMENT OF PATIENTS IN COMA |  |
| --- | --- |
| On average, how many coma patients do you treat per month? Specifically, patients in coma (based on the definition given above) *for ≥1 hour?* | 0, 1-5, 6-10, 11-15, 16-30, >30 |
| On average, how many coma patients do you treat per month? Specifically, patients in coma (based on the definition given above) *for ≥24 hours?* | 0, 1-5, 6-10, 11-15, 16-30, >30 |
| On average, how many coma patients do you treat per month? Specifically, patients in coma (based on the definition given above) *for ≥7days?* | 0, 1-5, 6-10, 11-15, 16-30, >30 |
| How many *pediatric* coma patients (aged<18 years) do you treat per month? Specifically, patients with absence of arousal and of awareness for ≥1 hour? | 0, 1-5, 6-10, 11-15, 16-30, >30 |
| Who is primarily responsible for performing the neurological exam in a critically ill comatose patient? | Attending physician  Neuro Intensivist  Medical Intensivist  Surgical Intensivist  Neurologist  Advanced Practice Provider  Other (specify) |
| On average, how often does this individual perform the neurological exam, assuming there are no acute neurological changes? | Upon admission and every hour  Upon admission and every 2 hours  Upon admission and every 4 hours  Upon admission and every 8 hours  Upon admission twice daily  Upon admission once daily  Other (specify) |
| How often do other members of the healthcare team (for example, nurses) perform a neurological exam, assuming there are no acute neurological changes? | Upon admission and every hour  Upon admission and every 2 hours  Upon admission and every 4 hours  Upon admission and every 8 hours  Upon admission twice daily  Upon admission once daily  Other (specify) |
| What pharmacological interventions to stimulate arousal among your comatose patients do you use in the MANAGEMENT of patients with prolonged coma (>24 hours)?  Check all that you routinely use as part of your management. | Sedation vacation  Sedation reversal (e.g. flumazenil, naloxone, naltrexone)  Antidote for drug or illicit drug overdose  Amantadine  Amphetamine/dextroamphetamine (Adderall)  Levodopa  Ambien  Dopamine agonist (e.g. pramipexole, ropinirole)  Methylphenidate (Concerta, Ritalin)  Modafanil (Provigil)  Osmotic therapy - hypertonic saline  Osmotic therapy - mannitol  Electrolyte/endocrine correction  IVIG  Plasma exchange/plasmapheresis  Steroids  Other (please specify) |
| What non-pharmacological interventions to stimulate arousal do you use in the MANAGEMENT of patients with prolonged coma (>24 hours) among your comatose patients?  Check all that you routinely use as part of your management. | Vagal nerve stimulation  Deep brain stimulation  Transcranial magnetic stimulation  Median nerve stimulation  Transcranial direct current stimulation  Sensory stimulation (includes deep pressure)  Other (please specify) |
| Do any of the following personnel routinely care for comatose patients in your institution? (check all that apply) | Physical therapist  Occupational therapist  Music therapist  Nutritionist/Dietician  Acupuncturist  Physiatrist  Other (please specify)  None of these |
| Please indicate the common rehabilitation trajectory for patients with coma on your unit: | Rehabilitation services and units available within our health system and most patients transfer to these services and units.  Our unit partners with a single rehabilitation center/unit and most of our patients transfer to this center/unit.  Our unit does not officially partner with one center/unit, instead our patients are transferred to a variety of different centers/units. |
| What is the most common discharge disposition for comatose patients who survive hospitalization in your institution? | Home  Home with services  Long term acute care hospital (LTACH)  Skilled Nursing Facility (SNF, nursing home)  Acute inpatient rehab facility  Subacute rehab facility  Other (please specify): |

| SECTION 5: ATTITUDES TOWARD PROGNOSIS |  |
| --- | --- |
| Please rank the top 3 (1^st^, 2^nd^, 3^rd^) most important elements you utilize for prognostication in comatose patients : | Age  Co-morbidities  Baseline functional status  Frailty scores  Etiology of Coma  Neurological Exam  Imaging results  Biomarkers  EEG findings  fMRI findings  Other (specify) |
| Does your unit have a formal policy or protocol for withdrawal of life sustaining therapies among patients with coma? | No  Yes  Don’t know |
| Is there a minimum assessment time before withdrawal of life sustaining therapies is considered among patients with coma in your unit? | No  Yes  If yes, please indicate how long:  Varies according to cause of coma |
| Do you think it is appropriate to withdraw life-supporting treatment within 72 hours of injury/illness? | No  Yes  Yes, but only for some causes of coma. Specify causes_____  If yes, in what percentage of comatose patients:  >90%, 75-90%, 50-74%, <50%  If yes, at what level of confidence? >90%, 75-90%, 50-  74%, <50%  Not sure |
| Do you believe it is possible to accurately predict poor long-term outcome (defined as vegetative state or severe disability) within 72 hours of injury/illness? | No  Yes  Yes, but only for some causes of coma. Specify causes_____  If yes, in what percentage of comatose patients  (check one): >90%, 75-90%, 50-74%, <50%  If yes, at what level of confidence (check one)? >90%,  75-90%, 50-74%, <50%  Not sure |
| When discussing prognosis with caregivers of patients who have had a disorder of consciousness for ≤ 28 days, do you believe that clinicians must avoid statements that suggest these patients have a universally poor prognosis? | No  Yes  Not sure |
| Is an ethicist involved in discussions about withdrawal of life sustaining therapies among patients with coma in your unit? | No  Yes, all of the time  Yes, in some cases, most of the time  Yes, in some cases, some of the time  Yes, in some cases, but rarely |
| What areas of coma research focus do you feel are most important/urgent? Please rank top 3 (1^st^, 2^nd^, 3^rd^ most important) | Epidemiology Pathophysiology/mechanisms of coma Diagnostics Treatment Prognostication Ethics |
| Are you willing to participate in any future studies?  *Indicate (yes/no/not sure) for each of the following:* | Annual surveys regarding coma launched on World Coma Day  A prospective observational trial including all comatose patients admitted to your ICU for a period of 3-6 months  A prospective observational trial including all comatose patients admitted to your ICU for a period of >6-12 months |
| Would you be able to follow up your patients?  Indicate (yes/no/not sure) for each of the following: | By telephone at 3 months  By telephone at 6 months  By telephone at 12 months  In person at 3 months  In person at 6 months  In person at 12 months  By video at 3 months  By video at 6 months  By video at 12 months |
| Please indicate if there is other information or management practices for patients with coma in your unit that you would like to share: | Free text |

**Supplemental Table 4**

**Title: Demographic characteristics of respondents**

| **Characteristics** | N, (%) |
| --- | --- |
| **Nationality (n = 258)** |  |
| US | 141 (55) |
| Europe | 53 (21) |
| Asia | 43 (17) |
| Latin America | 10 (4) |
| Africa | 7 (3) |
| Australia | 3 (1) |
| New Zealand | 1 (0.4) |
| **Years in practice (N=258)** |  |
| 0-5 | 56 (22) |
| 6-10 | 50 (19) |
| 11-15 | 40 (16) |
| 16-20 | 37 (14) |
| 21-30 | 44 (17) |
| >30 | 31 (12) |
| **Occupation (N=257)** |  |
| Physician | 213 (83) |
| Nurse practicioner | 11 (4) |
| Nurse | 8 (3) |
| Pharmacist | 8 (3) |
| PA | 2 (1) |
| Other | 15 (6) |
| **Affiliation (department) (N=258)** |  |
| Neurology | 120 (47) |
| Critical Care | 65 (25) |
| Neurosurgery | 20 (8) |
| Anesthesia | 15 (6) |
| Physical Medicine and Rehabilitation | 11 (4) |
| Pharmacy | 7 (3) |
| Nursing | 5 (2) |
| Other | 5 (2) |
| Emergency medicine | 4 (2) |
| Surgery | 3 (1) |
| Pediatrics | 3 (1) |
| **Subspeciality (N=252)** |  |
| Neurocritical Care | 193 (77) |
| Stroke | 46 (18) |
| Critical care medicine | 45 (18) |
| Anesthesia/critical care | 27 (11) |
| General neurology | 19 (8) |
| Trauma surgery | 16 (6) |
| Physical Medicine and Rehabilitation | 12 (5) |
| Surgical critical care | 10 (4) |
| General neurosurgery | 9 (4) |
| Vascular neurosurgery | 8 (3) |
| Pulmonary critical care | 8 (3) |
| Cardiac/cardiothoracic surgery critical care | 7 (3) |
| Endovascular/interventional | 4 (2) |
| Other | 21 (8) |
| **ICU Nurse to patient ratio (N=256)** |  |
| 1:1 | 34 (13) |
| 1:2 | 174 (68) |
| 1:3 | 27 (11) |
| 1:4 | 8 (3) |
| Other | 13 (5) |

**Supplemental Table 5**

**Title: Agreement to the definition of coma (N=238)**

|  | **Agreement** | **Neutral/Disagreement** | **Total** | **p-value** |
| --- | --- | --- | --- | --- |
| **Continents** | N=153 | N=85 | N=238 |  |
| Europe | 29 (19) | 21 (25) | 50 (21) |  |
| US | 90 (59) | 41 (48) | 131 (55) |  |
| New Zealand | 0 | 1 (1) | 1 (1) |  |
| Latin America | 5 (3) | 5 (6) | 10 (4) |  |
| Africa | 5 (3) | 2 (2) | 7 (3) |  |
| Asia | 21 (14) | 15 (18) | 36 (15) |  |
| Australia | 3 (2) | 0 | 3 (1) | 0.317 |
| **Number of years in practice** |  |  |  |  |
| 0-5 | 31 (20) | 19 (22) | 50 (21) |  |
| 6-10 | 29 (19) | 19 (22) | 48 (20) |  |
| 11-15 | 24 (16) | 14 (17) | 38 (16) |  |
| 16-20 | 20 (13) | 11 (13) | 31 (13) |  |
| 21-30 | 28 (18) | 14 (17) | 42 (18) |  |
| >30 | 21 (14) | 8 (9) | 29 (12) | 0.926 |
| **Area of practice** |  |  |  |  |
| Urban | 134 (88) | 74 (87) | 208 (87) |  |
| Suburban | 17 (11) | 7 (8) | 24 (10) |  |
| Rural | 2 (1) | 3 (4) | 5 (2) |  |
| Other, specify | 0 | 1 (1) | 1 (1) | 0.316 |
| **Practice setting** |  |  |  |  |
| Academic/Teaching hospital | 138 (90) | 75 (88) | 213 (89) |  |
| Non-teaching hospital | 4 (3) | 3 (4) | 7 (3) |  |
| Public non-academic hospital | 1 (1) | 2 (2) | 3 (1) |  |
| Private non-academic hospital | 5 (3) | 4 (5) | 9 (4) |  |
| District or Government Hospital | 1 (1) | 1 (1) | 2 (1) |  |
| Other, specify | 4 (3) | 0 | 4 (2) | 0.531 |
| **Occupation** |  |  |  |  |
| Physician | 132 (86) | 69 (82) | 201 (85) |  |
| NP | 8 (5) | 3 (4) | 11 (5) |  |
| PA | 0 | 1 (1) | 1 (1) |  |
| nurse | 4 (3) | 2 (2) | 6 (3) |  |
| pharmacist | 1 (1) | 4 (5) | 5 (2) |  |
| Other, specify | 8 (5) | 5 (6) | 13 (6) | 0.248 |
| **What is your primary department affiliation** | |  |  |  |
| Neurology | 68 (44) | 45 (53) | 113 (48) |  |
| Neurosurgery | 16 (11) | 4 (5) | 20 (8) |  |
| Surgery | 2 (1) | 1 (1) | 3 (1) |  |
| Anesthesia | 10 (7) | 4 (5) | 14 (6) |  |
| Critical Care | 38 (25) | 22 (26) | 60 (25) |  |
| Physical Medicine and Rehabilitation | 9 (6) | 1 (1) | 10 (4) |  |
| Pediatrics | 3 (2) | 0 | 3 (1) |  |
| Emergency medicine | 3 (2) | 1 (1) | 4 (2) |  |
| Nursing | 2 (1) | 2 (2) | 4 (2) |  |
| Pharmacy | 1 (1) | 3 (4) | 4 (2) |  |
| Other, specify | 1 (1) | 2 (2) | 3 (1) | 0.261 |
| **Cardinal features of coma** |  |  |  |  |
| Cardinal feature GCS<9 | 98 (64) | 50 (59) | 148 (62) | 0.486 |
| Absence of wakefulness | 126 (82) | 65 (77) | 191 (80) | 0.309 |
| No eye opening | 80 (52) | 45 (53) | 125 (53) | 1.000 |
| No visual pursuit of objects, fixation or saccade to stimuli | 84 (55) | 32 (38) | 116 (49) | **0.015** |
| Inability to follow commands (excluding aphasic patients) | 95 (62) | 41 (48) | 136 (57) | **0.041** |
| No intelligible speech or recognizable gesture | 73 (48) | 29 (34) | 102 (43) | 0.055 |
| Failure to respond purposefully to visual, verbal or tactile stimuli based on clinical exam | 99 (65) | 46 (54) | 145 (61) | 0.128 |
| No evidence of cognitive motor dissociation (i.e. the covert ability to follow commands) based on exam, neurophysiological studies or functional imaging | 68 (44) | 33 (39) | 101 (42) | 0.415 |

**Supplemental Figure 1**

**Title:** Relative distribution of coma patients treated per month defined by the duration of coma (N= 225/258 responses)

**Survey question:** *On average, how many coma patients do you treat per month?*

*Data on the y-axis are given in percentages.*

**Supplemental Figure 2**

**Title**: Most common etiologies of coma weighted by the 5 most common causes based on the participant definition of coma

**Survey question:** *Rank the top 5 most common etiologies of coma that you encounter in your institution based on your definition of coma*

Bars represent the selection of etiologies based on the most common (blue), second most common (red), third most common (grey), fourth most common (yellow), fifth most common (green) etiology of coma. Data are given in percentage and weighted based on the grading of respondents, relative to the most common etiology (intracerebral hemorrhage). The answers were weighted based on the most common (multiplied by 5), the second most common (multiplied by 4), the third most common (multiplied by 3), the fourth most common (multiplied by 2) and the 5th most common etiology (multiplied by 1).

**Supplemental Figure 3**.

**Title**: Discharge disposition for comatose patients (N=230/258)

Survey question: What is the most common discharge disposition for comatose patients who survive hospitalization in your institution?
